# Supplementary material for: Metatranscriptomic Analysis of Oil-Exposed Seawater Bacterial Communities Archived by an Environmental Sample Processor (ESP)
Source: Microorganisms. 2020 May 15;8(5):744. doi: 10.3390/microorganisms8050744 (PMC7284936; doi:10.3390/microorganisms8050744)
Supplement: Supplementary file 1 [file microorganisms-08-00744-s001.zip › Supplementary Materials-revised.pdf]

Supplementary Materials

Table 1. Sample filtration volume and RNA quantification of samples sequenced in this study.

	T0	T4C	T4O	T7C	T7O
Sample filtration volume (ml)	1000	700	700	700	700
RNA concentration (ng/µl)	57	216	201	78	177
Total RNA sent (µg)	2.3	8.6	8.0	3.1	7.0

Table 2. Statistics of sequence processing.

	**T0**	**T4C**	**T4O**	**T7C**	**T7O**
Raw reads (Million)	37.1	52.5	54.9	49.9	48.7
Total bases (Gb)	2.8	3.96	4.15	3.76	3.67
Read length (bp)	75	75	75	75	75
Quality filtered reads	17,816,080	28,530,130	29,020,210	27,545,407	23,999,441
Number of assembled contigs			628,164		
Number of predicted CDS			674,434		
Reads mapped back to contigs	6,185,558	19,813,474	19,903,786	18,641,091	17,069,785
Reads mapped back to CDS	4,738,252	16,890,778	16,906,056	15,901,564	14,358,262

Table 3. Uploaded as an Excel Worksheet. Contains: Table S3.1 Total hydrocarbon content and Table S3.2 Concentration of aromatic hydrocarbons.

Table 4. Diversity metrics calculated on family level from the RNA sequencing (RNAseq) and amplicon sequencing (16S) datasets. *H* – Shannon index and *J* – Pielou's evenness.

	**T0**		**T4C**		**T4O**		**T7C**		**T7O**	
	RNAseq	16S	RNAseq	16S	RNAseq	16S	RNAseq	16S	RNAseq	16S
H	2.108	n.a.	1.935	1.971	1.978	1.740	1.973	1.934	1.979	1.714
J	0.418	n.a.	0.401	0.484	0.406	0.430	0.405	0.485	0.409	0.420

Table 5. Uploaded as an Excel Worksheet. Contains: Table S5.1. Taxonomic classification of microbial community in seawater exposed to oil and seawater control observed at the start of exposure (T0), at the day 4 and at the last day of exposure (day 7) determined by metatranscriptomic and 16S rRNA amplicon, Table S5.2. Fold change difference between the oil and control samples observed at day 4 and day 7 after exposure, Table S5.3. Taxonomic classification of microbial community at the family level, Table S5.4. Taxonomic classification of microbial community at the genus level and Table S5.5. Taxonomic classification of microbial community determined by 16S amplicon sequencing analysis.

Table 6. Uploaded as an Excel Worksheet. Contains: Table S6.1. Blast analysis of the predicted CDS against the NCBI protein (nr) database and Table S6.2. Blast analysis of the predicted CDS against the KEGG database.

Table 7. Uploaded as an Excel Worksheet. Contains: Summary of results obtained with differentially abundant CDS that were significantly upregulated on seawater with oil.

Table 8. Uploaded as an Excel Worksheet. Contains: Table S8.1. KEGG pathway analysis of upregulated CDS (n=4,503) that were assigned K numbers (1,037), Table S8.2. Upregulated CDS (n=2,564) that were assigned K numbers (544) but were not mapped on KEGG pathway maps, Table S8.3. TPM counts of upregulated CDS that were assigned functions using KEGG PATHWAY/BRITE analysis and visualized on Figure 7 and Table S8.4. TPM counts and taxonomic annotation of selected upregulated KEGG genes related to oil degradation.

Table 9. Uploaded as an Excel Worksheet. Contains: Table 9.1. TPM counts for viral hits (LCA family level), Table 9.2. TPM counts for viral hits (family only), Table 9.3. TPM counts for viral hits (LCA species level), Table 9.4. TPM counts for viral hits (species only) and Table 9.5. TPM counts for phage hosts.

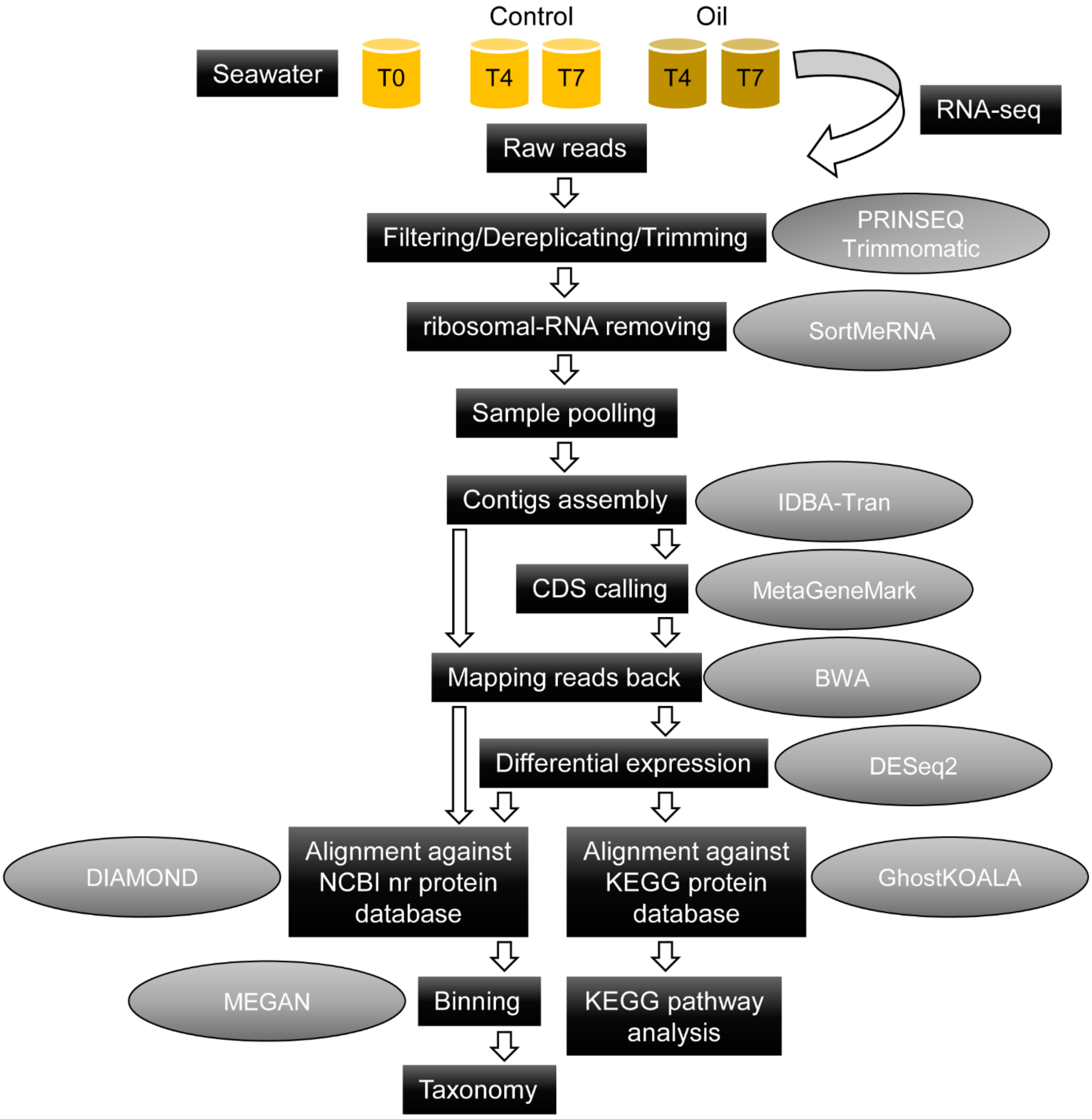

Figure S1. Flowchart of the bioinformatic analysis.

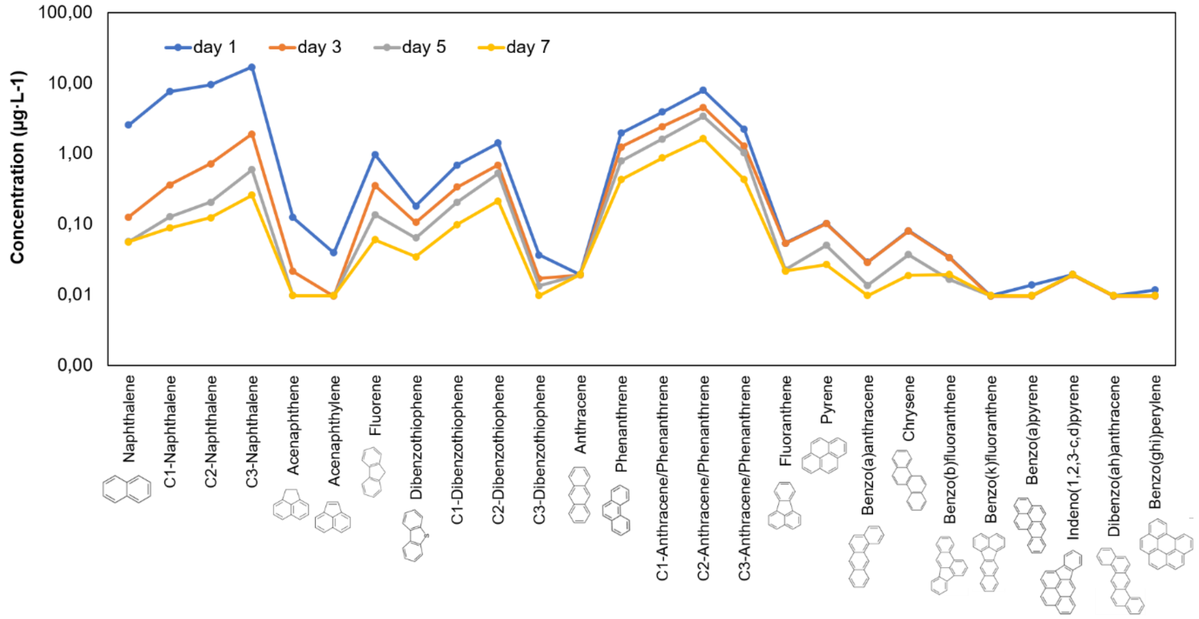

Figure S2. Concentrations of individual polycyclic aromatic hydrocarbons measured on day1, 3, 5 and 7 of the experiment–shown on a logarithmic scale due to orders of magnitude differences between the different compounds.

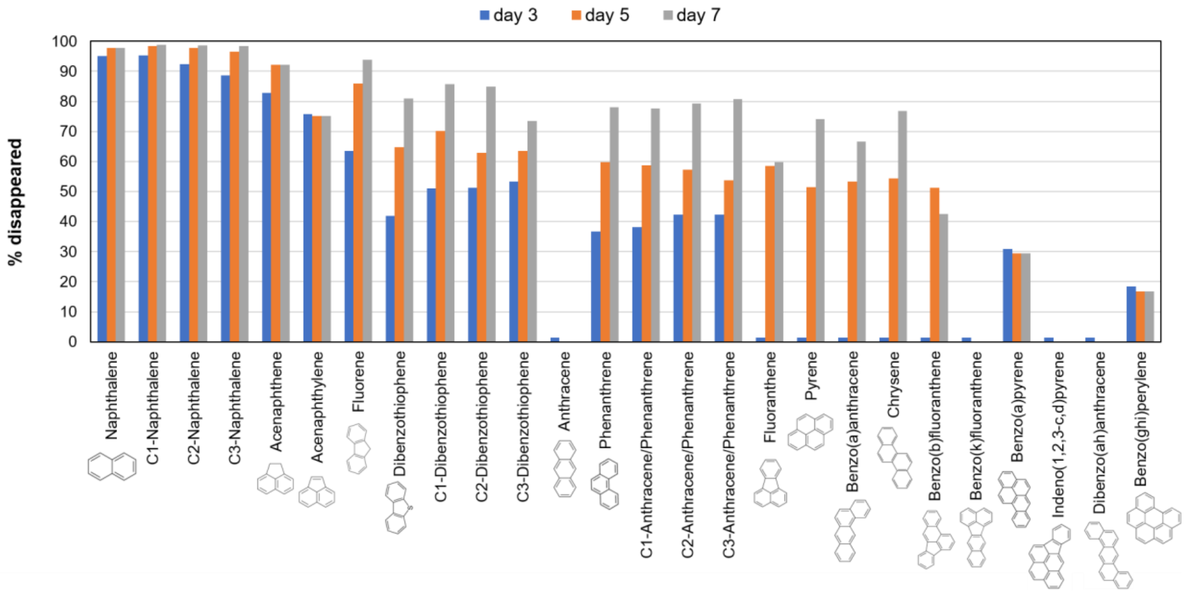

Figure S3. Percent disappeared values for each individual polycyclic aromatic hydrocarbon – day 1 values were used as reference.

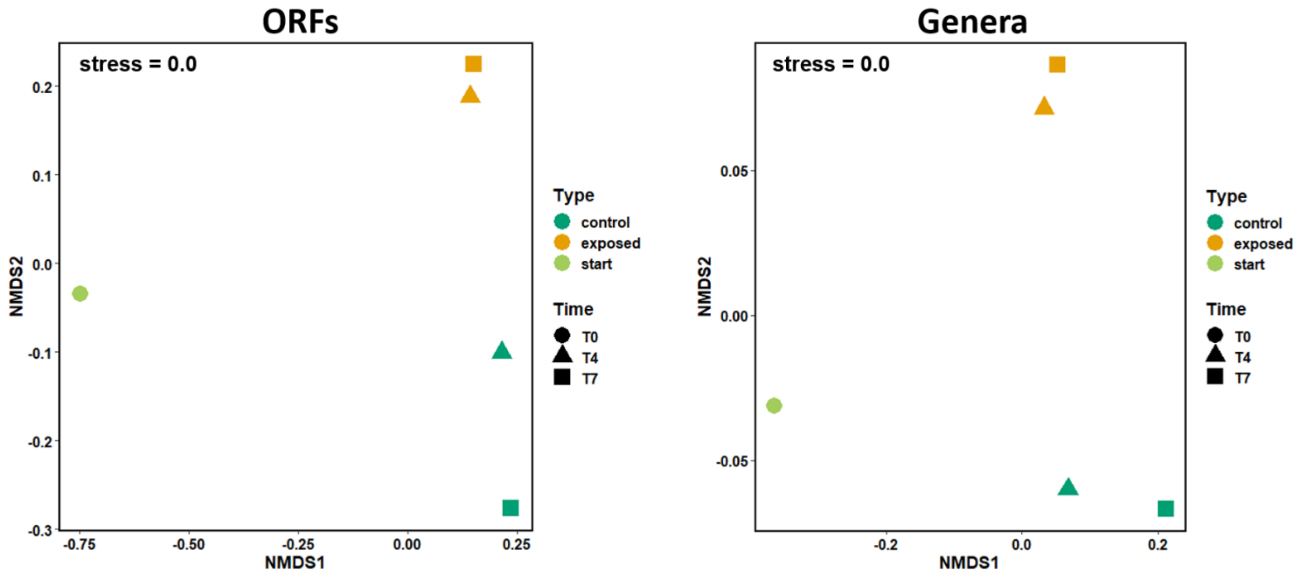

Figure S4. Non-metric multidimensional scaling (NMDS) plots on Bray-Curtis dissimilarities of ORFs (Left) and genera (Right).

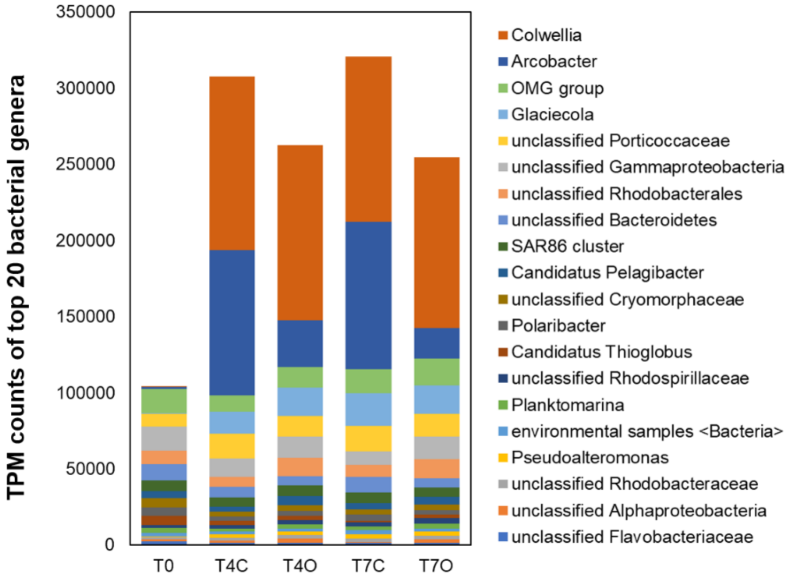

Figure S5. Genus level composition of contigs. Cumulative TPM counts of the top 20 (based on sum TPM across the 5 samples) genera.

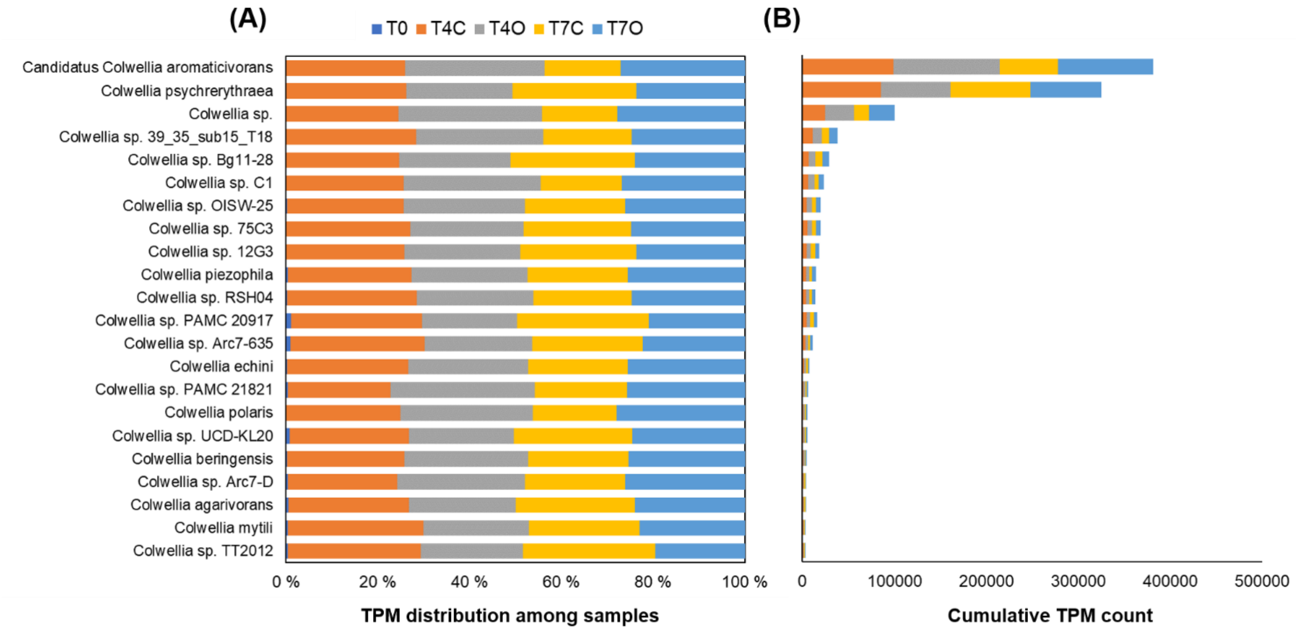

Figure S6. Species level composition of Colwellia contigs. A: TPM distribution of the different Colwellia species among the 5 samples. B: Cumulative TPM counts of the various Colwellia species.
